# Effect of Secondary Phase Precipitation on the Corrosion Behavior of Duplex Stainless Steels

**DOI:** 10.3390/ma7075268

**Published:** 2014-07-22

**Authors:** Kai Wang Chan, Sie Chin Tjong

**Affiliations:** Department of Physics and Materials Science, City University of Hong Kong, Tat Chee Avenue, Kowloon, Hong Kong, China; E-Mail: kaiwchan8-c@my.cityu.edu.hk

**Keywords:** duplex stainless steel, sigma phase, spinodal decomposition, nitride, intergranular corrosion, critical pitting temperature, stress corrosion, welding

## Abstract

Duplex stainless steels (DSSs) with austenitic and ferritic phases have been increasingly used for many industrial applications due to their good mechanical properties and corrosion resistance in acidic, caustic and marine environments. However, DSSs are susceptible to intergranular, pitting and stress corrosion in corrosive environments due to the formation of secondary phases. Such phases are induced in DSSs during the fabrication, improper heat treatment, welding process and prolonged exposure to high temperatures during their service lives. These include the precipitation of sigma and chi phases at 700–900 °C and spinodal decomposition of ferritic grains into Cr-rich and Cr-poor phases at 350–550 °C, respectively. This article gives the state-of the-art review on the microstructural evolution of secondary phase formation and their effects on the corrosion behavior of DSSs.

## 1. Introduction

Stainless steels are an important class of engineering alloys that have found widespread applications from domestic home appliances to structural components in aerospace industries. In particular, duplex stainless steels (DSSs) with austenitic (fcc) and ferritic (bcc) grains possess beneficial combinations of these two phases [1,2,3,4,5,6]. DSSs exhibit greater toughness and better weldability than ferritics. Compared with austenitic grades, DSSs have higher resistance to pitting and stress corrosion cracking. Accordingly, they are widely used in various chemical, petrochemical, food, power, transportation, pulp and paper industries as well as oil refineries.

The high corrosion resistance of DSSs derives from their high Cr content in combination with substantial additions of Mo, Ni and N. Chromium contributes to the corrosion resistance of stainless steels by forming protective Cr-oxide/hydoxide in the passive film [7]. The presence of Mo within the passive film of DSSs and synergistic effect between the oxides/hydroxides of Cr and Mo improve the film stability against pitting corrosion [8]. As recognized, Cr, Mo and Si alloying elements stabilize ferritic phase, while Ni and N are the γ-phase stabilizers [9]. N is an interstitial solid solution strengthener that increases the strength of DSSs [10]. It also increases the pitting resistance of DSSs and concentrates mainly at the metal-passive film interface. Manganese stabilizes the austenite, but it is not effective as Ni in stabilizing the γ-phase. Therefore, N is added with Mn simultaneously to DSSs to balance a decrease in the Ni content [11,12,13,14,15]. Cu addition is beneficial for enhancing the resistance of DSSs in non-oxidizing solutions. Its content is limited to ~2.5% since higher content reduces hot ductility [2]. The ferrite/austenite ratio in DSSs must be close to 50:50 by adding appropriate alloying elements for achieving desired microstructures and mechanical properties. A wide range of DSSs with different alloying elements has been developed and commercialized. The most widely used DSS is the standard 2205 grade. To further improve corrosion resistance, DSSs with higher Cr content such as 25% Cr grade (UNS S32550 and S32950), superduplex (UNS S32750), and hyperduplex (S32707 and S33207) are produced [16,17]. Superduplex (UNS S32750) is a highly alloyed grade having good chloride resistance and high mechanical strength. Recently, hyperduplex grade steels with higher amounts of alloying elements and N content up to 0.5 wt% are particularly suitable for use in severe marine solutions. The high Cr, Mo and N contents render them with excellent corrosion resistance, high strength and good formability for extrusion into seamless tubes for subsea umbilical applications [17]. Table 1 lists typical chemical compositions of commercial DSSs. The chemical compositions of DSSs play a dominant role in controlling their microstructures and properties. The processing, property and microstructure of DSS is rather complex. A comprehensive review on the processing-structural property of DSSs can be found elsewhere [1,2,3,4,5,6], and beyond the scope of this article.

**Table 1:** Chemical compositions (wt%) of duplex stainless steels (Fe content: balance).

UNS No.	EN No.	Common Name	C, max	Cr	Ni	Mo	Mn	Si	Co	Cu	N
S31803	1.4462	2205	0.03	21–23	4.5–6.5	2.5–3.5	2.0	1.0	–	–	0.08–0.2
S32205	1.4462	2205	0.03	22–23	4.5–6.5	3.0–3.5	2.0	1.0	–	–	0.14–0.2
S32550	1.4507	255	0.04	24–27	4.5–6.5	2.9–3.9	1.5	1.0	–	1.5–2.5	0.10–0.25
S32950	–	7Mo Plus	0.03	26–29	3.5–5.2	1.0–2.5	2.0	0.6	–	–	0.15–0.35
S32750	1.4410	2507	0.03	24–26	6.0–8.0	3.0–5.0	1.2	0.8	–	0.5	0.24–0.32
S32707	–	SAF 2707HD	0.03	27	6.5	4.8	1.0	0.3	1.0	–	0.4
S33207	–	SAF 3207HD	0.03	32	7	3.5	1.0	0.3	–	–	0.5
S32304	1.4362	2304	0.03	21.5–24.5	3.0–5.5	0.05–0.6	2.50	1.0	–	0.05–0.6	0.05–0.2
S32101	1.4162	LDX 2101	0.04	21–22	1.35–1.7	0.1–0.8	4.0–6.0	1.0	–	0.1–0.8	0.2–0.25

DSSs undergo microstructural changes during heat treatment, welding process and prolonged engineering service at high temperatures. Several undesirable precipitates such as carbides, nitrides, intermetallic phases (sigma and chi), and Cr-rich α’ phase can induce in DSSs upon exposure to 950–400 °C temperature range [1,2,3,4,5,6,16]. Sigma phase is preferentially nucleated at the ferrite-austenite and ferrite-ferrite boundaries of DSSs at 700–950 °C [18,19]. Is formation depletes Cr and Mo at these regions, resulting in intergranular corrosion (IGC) and pitting corrosion [18,19,20,21,22,23]. Chromium carbides (Cr_23_C_6_) may form at the DSSs’ grain boundaries upon heating at ~550–750 °C. The risk of Cr_23_C_6_ precipitation in commercial DSSs is rather small since their C content is kept at a maximum value of 0.03 wt%. At lower temperature regime of 350–550 °C, the α- phase transforms into Cr-rich (α’) and Cr-poor (α) phases via a spinodal decomposition [24]. The Cr-rich α’ precipitates reduces the impact toughness and corrosion resistance of DSSs significantly [25,26]. The spinodal decomposition is more pronounced at 475 °C, commonly referred to as the 475 °C embrittlement. This article focuses exclusively on the deleterious effect of these undesired phases on the corrosion resistance of DSSs.

## 2. Secondary Phase Formation

### 2.1. Sigma, Chi and Nitride Phases

The literatures from 1983 to 2009 have reported the structures, morphologies and techniques for detecting intermetallic compounds and spinodal decomposed phases in details [1,2,3,4,5,6], with particular emphasis on the formation, characteristics and morphology of such precipitates and their adverse effects on the mechanical properties of DSSs [5,6]. The readers may refer to the literatures for further details [1,2,3,4,5,6]. Thus, we briefly presented such topics herein. Sigma phase exhibits tetragonal structure containing about 30% Cr, 4% Ni and 7% Mo [2]. The austenitic phase of DSSs exhibits closed-packed, face centered cubic structure. The precipitation reaction of σ-phase in the γ-phase is sluggish due to a slow diffusivity of solute atoms in this phase. As just mentioned, Cr, Mo and Si are ferrite formers that promote the σ-phase precipitation in DSSs aged at 700–950 °C. The diffusion rates of these elements in ferrite are much faster than in the austenite. As the precipitation continues, Cr and Mo diffuse to the σ-phase, leading to a depletion of these elements in ferrite, especially Mo content. Therefore, Mo from the inner region of ferrite diffuses to the σ-phase. Sigma phase nucleates preferentially at the α- and α-γ boundaries, then grows into the adjacent ferritic grains. Mo is the main element controlling secondary phase precipitation. It is noted that the χ-phase (bcc; Fe_36_Cr_12_Mo_10_) containing about 25% Cr, 3% Ni and 14% Mo also forms between 700 and 900 °C, but in much smaller amounts [2]. The χ-phase contains even more Mo than the σ-phase, and nucleates in the early stage of aging due to the low interfacial energy of highly coherent χ/α interface with a characteristic of cubic-to-cubic orientation relationship [2,27]. Generally, ferrite can transform to secondary phases via the following eutectoid reaction,

α (ferrite) → σ + χ + Cr_2_N (1)

Although the χ-phase forms earlier than the σ-phase, it transforms into the σ-phase after prolonged aging [27,28]. The growth of σ-and χ-phase precipitates further depletes Cr and Mo in ferrite. Consequently, ferrite phase with high Ni content becomes unstable and eventually transforms into secondary austenite (γ_2_). DSSs generally contain N up to 0.5 wt% for enhancing mechanical strength and pitting corrosion resistance (Table 1) Chromium nitride (Cr_2_N; hexagonal structure) can form in DSSs with higher N content, and in the fusion zone of DSSs during welding.

**Figure 1:**
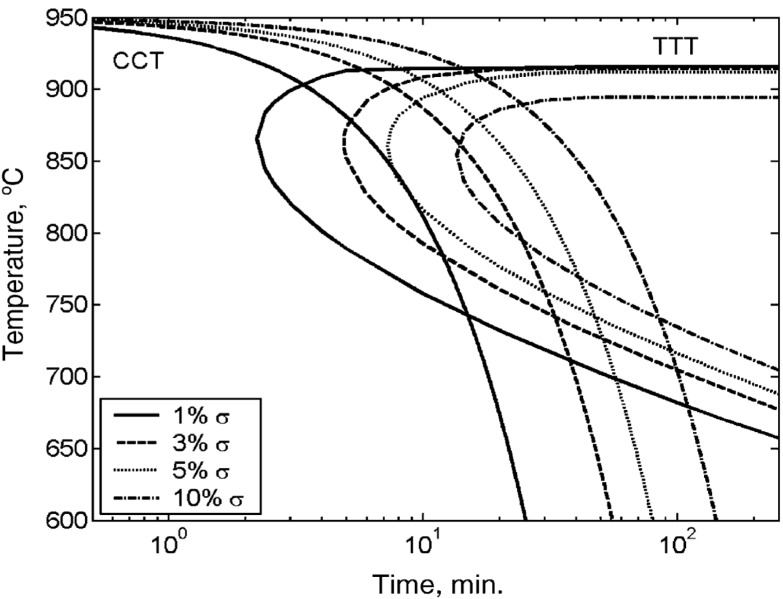
Time-temperature transformation (TTT) and continuous-cooling transformation (CCT) curves of duplex stainless steel (DSS) 2205 showing transformation of 1, 3, 5 and 10% σ-phase. The cooling rates are 0.23, 0.11, 0.07 and 0.04 °C/s, Reprinted with permission from [30]. Copyright 2007 Elsevier.

Time-temperature transformation (TTT) diagrams obtained from isothermal heat treatment followed by quenching, can be used to investigate the susceptibility of DSSs to the σ-phase formation. This diagram indicates the time for a phase to decompose into other phases isothermally at different temperatures. In contrast, continuous-cooling transformation (CCT) diagram displays the time for a phase to decompose into other phases continuously at different rates of cooling. Figure 1 shows typical TTT and CCT curves for DSS 2205 with different σ-phase contents [29]. Isothermal heat treatment at 865 °C for 134 s precipitates 1% σ-phase. This implies that the aging time at 865 °C should not exceed 134 s. The 1% σ content can be obtained at room temperature after continuous cooling with 0.23 °C/s at 940 °C and above. To avoid the formation of more than 1% σ, the cooling rate from the solution annealing temperature must exceed 0.23 °C/s.

Materials examination techniques are very important for the detection and identification of deleterious precipitates in DSSs. The structures of secondary phases can be determined with the X-ray diffraction (XRD), electron diffraction and electron backscattered diffraction (EBSD) techniques. The microstructural features of these phases in DSSs can be obtained using optical microscopy, scanning electron microscopy (SEM) and transmission electron microscopy (TEM) [27,30,31]. Combining with electron diffraction, TEM permits the examination of structural and morphological of localized region in a thin foil specimen at high magnifications. TEM is useful for characterizing the structures of fine precipitates such as σ- and χ- phases, especially in small amounts. However, the preparation of TEM thin film specimens is tedious, time consuming and difficult. EBSD involves the analysis of Kikuchi line bands formed by diffusively scattered electrons of individual crystals of a specimen. It provides easier identification of the crystal structure, grain orientation of unknown phases without the need for thin film sample preparation and enables observation of larger areas of a specimen. Recently, EBSD has been found to be very effective for detecting σ-phase formed in DSSs [32].

**Figure 2:**
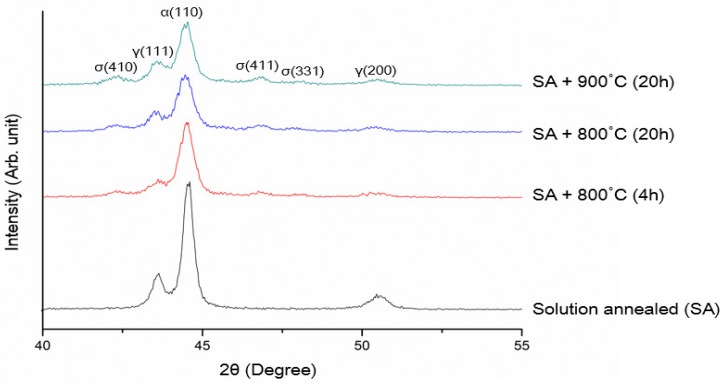
XRD patterns of solution-annealed DSS 2205, 4 h aged at 800 °C, 20 h aged at 800 °C and 20 h aged at 900 °C steel samples (CuK_α_ radiation, wavelength: 0.154 nm).

**Figure 3:**
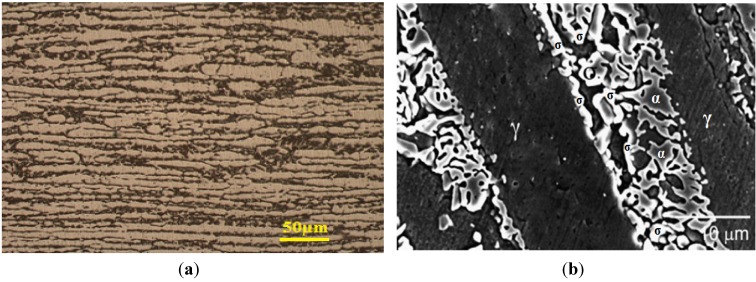
(**a**) Optical micrograph of DSS 2205 aged at 800 °C for 20 h. Dark phase is ferrite and bright phase is austenite; (**b**) Backscattered electron image of DSS 2205 aged at 875 °C for 20 min, reprinted with permission from [31]. Copyright 2009 Elsevier.

**Figure 4:**
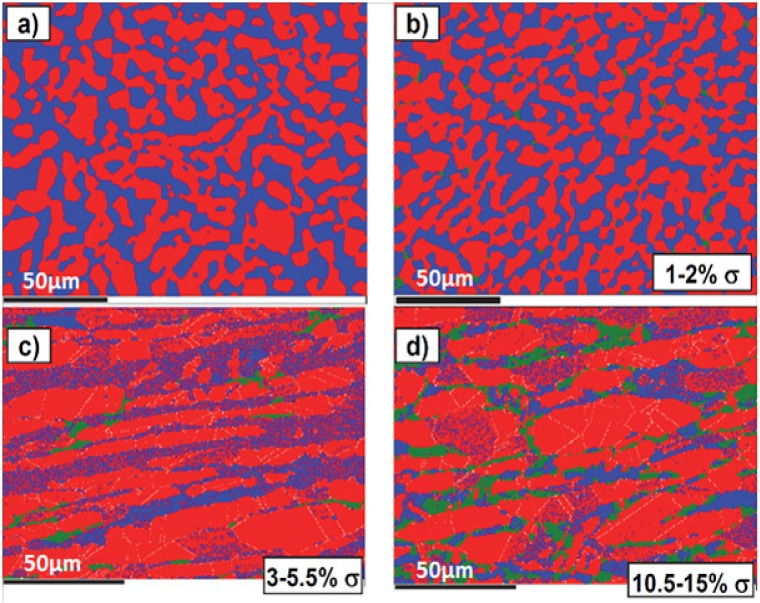
Electron backscattered diffraction (EBSD) phase maps for DSS 2205: (**a**) as-received; (**b**) aged at 900 °C for 30 min; (**c**) aged at 750 °C for 2 h and (**d**) aged at 750 °C for 5 h. Ferrite (blue), austenite (red), sigma (green), reprinted with permission from [32]. Copyright 2014 the IOP Publishing.

Figure 2 shows the XRD patterns of solution-annealed and aged DSS 2205 specimens. Solution annealing DSSs at high temperatures followed by water quenching is very effective to eliminate the σ-phase formation. The σ-phase peaks are observed in DSSs after aging at 800 and 900 °C. The typical solution temperature range is 1050–1080 °C. Above 1080 °C, e.g., at 1200 °C, unfavorable high α (62 vol%) and low γ-phase (38 vol%) content is produced [33]. The cooling rate from solution annealing temperature must exceed 0.23 °C/s to avoid the σ-phase formation as mentioned previously. The σ-phase with higher Cr and Mo contents than α- and γ-phases appears brighter in the back-scattered electron (BSE) image (Figure 3b) [31]. This figure reveals that the σ-phase forms preferentially at the α/γ and α/α boundaries, and penetrates into the α-phase. This phase can be clearly seen in the EBSD phase maps of aged DSS 2205, particularly after aging at 750 °C for 5 h (Figure 4) [32].

**Figure 5:**
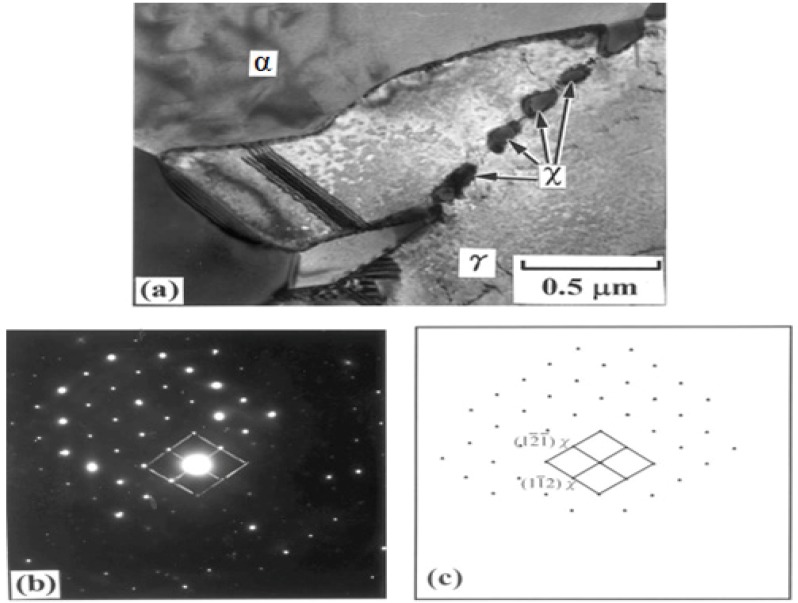
(**a**) TEM image of χ-phase of DSS 2205 aged at 750 °C for 10 min; (**b**) selected area electron diffraction (SAED) pattern with [5 3 1]_χ_ zone and (**c**) index diagram, reprinted with permission from [27]. Copyright 2002 Elsevier.

**Figure 6:**
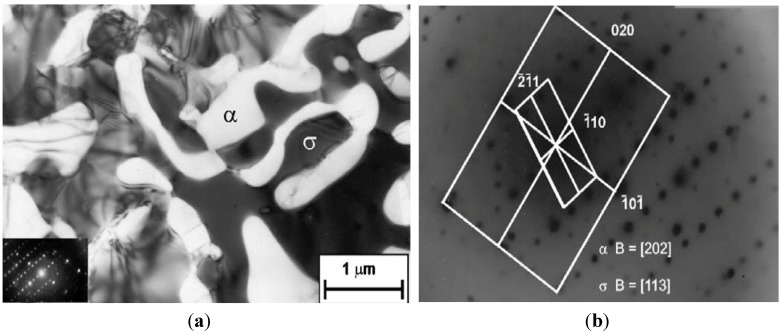
(**a**) TEM image showing σ-particle embedded in the α-phase matrix; (**b**) SAED pattern showing [202]_α_ and [113]_σ_ zones, reprinted with permission from [31]. Copyright 2006 Elsevier.

**Figure 7:**
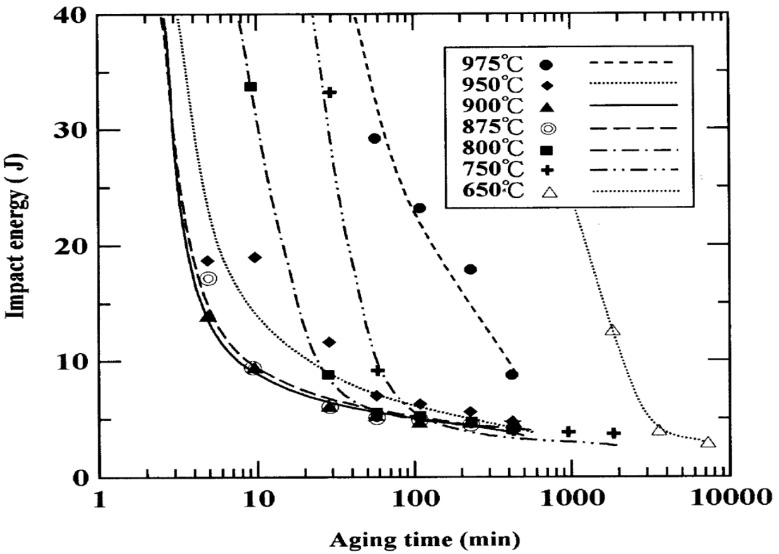
Effect of aging treatment on Charpy impact toughness of DSS 2205, reprinted with permission from [27]. Copyright 2002 Elsevier.

As mentioned earlier, bcc χ-phase forms in the earlier stage in the bcc ferrite of DSSs due to preferential cube-on-cube orientation relationship. The χ-phase then transforms to the σ-phase upon prolonged aging [27]. Figure 5a is the TEM image showing precipitation of χ-phase at the α-γ interface of DSS 2205 aged at 750 °C for 10 min only. The selected area electron diffraction (SAED) pattern and its index diagram are shown in Figure 5b,c, respectively. Michalska and Sozanska [31] reported that the χ and σ-phases precipitate preferentially at the α-γ interface and within the α-phase. The volume fraction and size of σ-phase increase with aging time. EDX results revealed that the χ- phase is highly enriched with Mo (15.95 wt%). Figure 6a shows the TEM image of DSS 2205 aged at 750 °C for 5 h. The SAED pattern is shown in Figure 6b. The σ-phase grows further into the α-phase after aging for 5 h because of high diffusivity of solute atoms at high temperatures. This phase is detrimental to the impact toughness of DSS 2205 (Figure 7).

**Figure 8:**
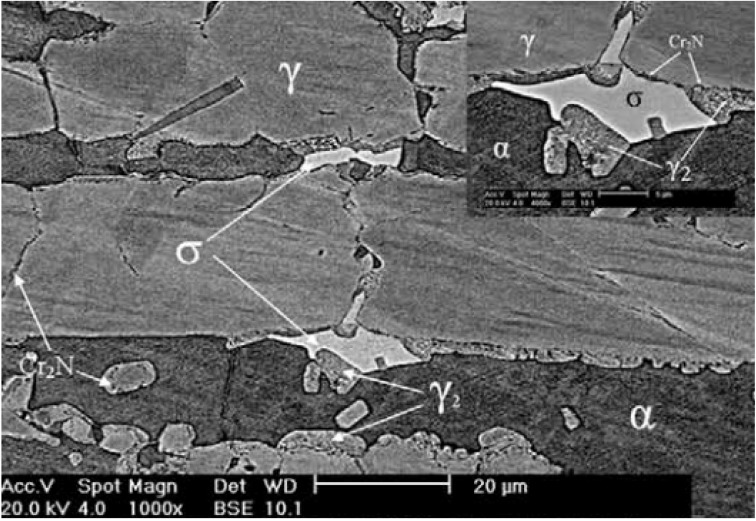
Backscattered electron image of UNS S32101 steel aged at 700 °C for 168 h, reprinted with permission from [36]. Copyright 2010 Elsevier.

For lean DSS S32101, the kinetics of σ-phase precipitation is much slower than S31803 due to its reduced Cr and extremely low Mo contents. Fine Cr_2_N particles nucleate at the α/α and α/γ boundaries by aging at 700 °C at the initial stage of aging (240 min) [34,35,36]. The Cr_2_N precipitation depletes Cr in the ferrite phase, resulting in the γ_2_ formation via a reaction α → Cr_2_N + γ_2_. Further aging to 168 h leads to the formation of a small amount of σ-phase adjacent to the Cr_2_N precipitates (Figure 8). Prolonged aging for 300 h yields an increase in the σ-phase volume fraction.

### 2.2. Spinodal Decomposition

**Figure 9:**
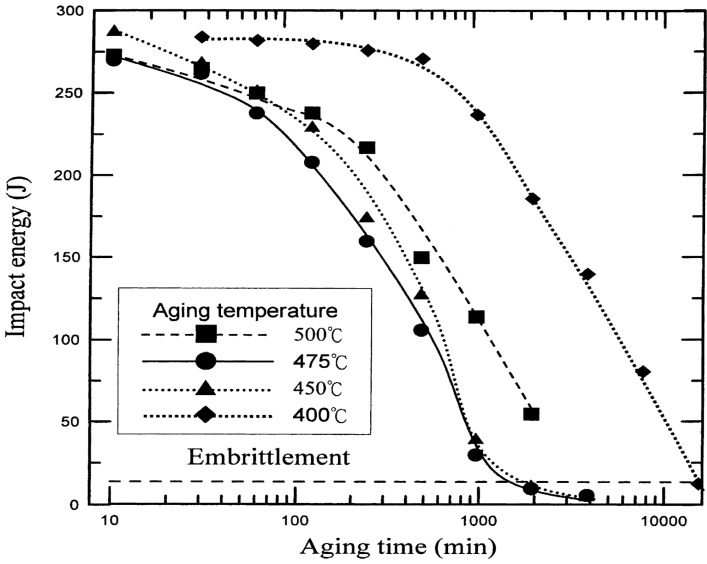
Impact energy *vs.* aging time for DSS 2205 treated at 400–500 °C, reprinted with permission from [38]. Copyright 2004 Elsevier.

**Figure 10:**
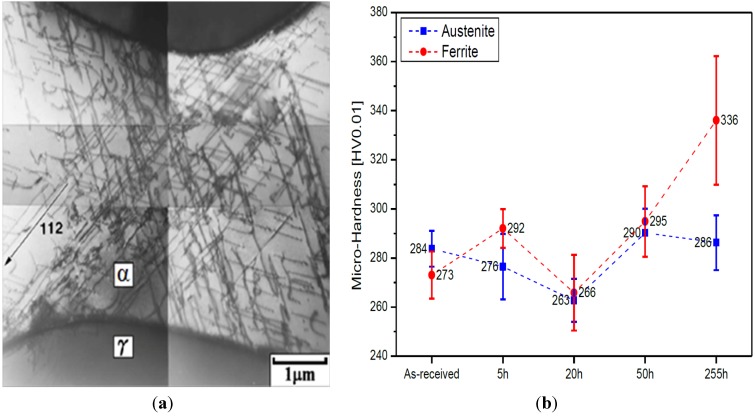
(**a**) Dislocation structure in DSS 2205 aged at 475 °C for 64 h, reprinted with permission from [38]. Copyright 2004 Elsevier; (**b**) Microhardness of ferritic and austenitic grains *vs.* aging time of DSS 2205 treated at 475 °C, reprinted with permission from [37]. Copyright 2013 Institute of Corrosion, UK.

**Figure 11:**
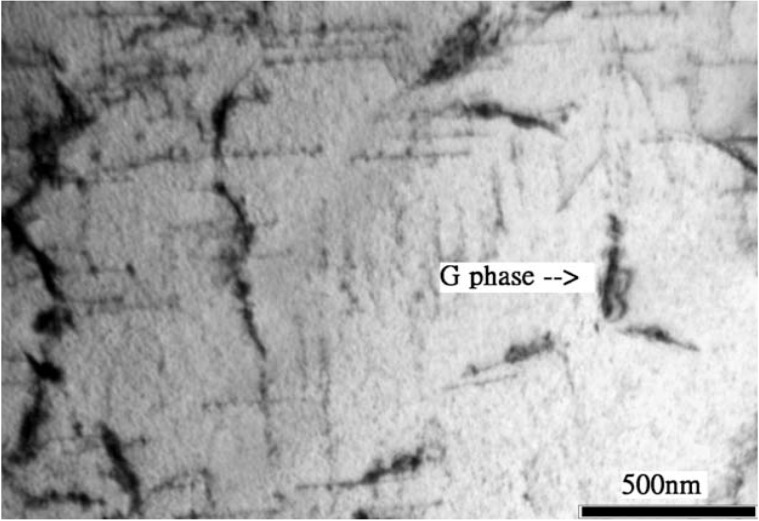
Development of dislocations and precipitation of G-phase in the 7MoPlus steel aged at 500 °C for 1744 h, reprinted with permission from [41]. Copyright 2010 Elsevier.

Spinodal decomposition of ferrite is originally found in ferritic Fe-Cr alloys heated at 280–500 °C due to the presence of a miscibility gap in the alloy system. Duplex stainless steels also suffer from the “475 °C embrittlement” associated with the precipitation of Cr-rich α’-phase and Fe-rich α-phase within ferritic grains. This decomposition occurs at 350–550 °C, with the most rapid formation at 475 °C [37,38,39]. Weng *et al.* [38] aged DSS 2205 at 400–500 °C for different times, and examined microstructures and mechanical properties of the aged specimens. Low-temperature aging treatment causes a significant reduction in the impact strength of DSS 2205, especially at 475 °C with aging times ≥1000 min (Figure 9). They also reported that the ferrite phase exhibits a modulated contrast, displaying the appearance of an orange-peel because of aging treatment. The mottled aspect is caused by compositional fluctuations associated with the formation of Cr-rich and Cr-poor phases. Furthermore, highly dense dislocations are created in the ferrite phase owing to a difference in thermal expansion coefficient between ferritic and austenitic grains upon cooling from the decomposition temperatures. The immobilization of dislocations hardens ferritic grains considerably (Figure 10) [37,38,39,40]. Prolonged aging treatment leads to the formation of G-phase (fcc; 25% Cr, 25% Ni, 4% Mo) along the dislocations in the ferrite phase (Figure 11) [2,41]. It is noted that TEM mottled contrast gives misleading indication for spinodally decomposed phases in DSSs. This is because the difference in lattice parameter between the Cr-rich and Fe-rich is very small. The Cr-rich phase is coherent with the alloy matrix. There is no clear interface between the precipitate and the matrix. Thus it is difficult to detect accurately the composition fluctuations in spinodally decomposed products using electron microscopy. However, atom probe technique is attractive to study the ultrafine scale phase separation and compositions in sub-nanometer scale. Some researchers used the atom probe technique in combination with field ion microscopy to elucidate this issue [42,43,44,45,46].

**Figure 12:**
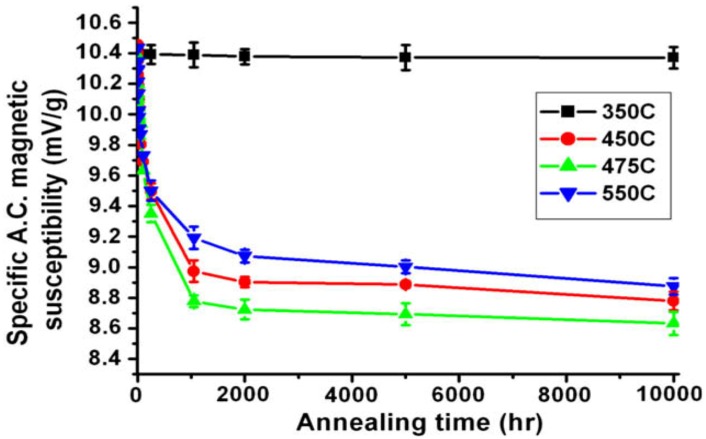
Variation of magnetic susceptibility with aging time of 7MoPlus steel treated at 350, 450, 475 and 550 °C, reprinted with permission from [41]. Copyright 2010 Elsevier.

Recently, Lo and Lai [41] demonstrated that a.c. magnetic susceptibility is an effective tool for detecting spinodal decomposition of the 7MoPlus steel (UNS S32950). Figure 12 shows magnetic susceptibility *vs.* aging time for 7MoPlus. Magnetic susceptibility is a dimensionless quantity that describes the magnetization of a material in response to an applied magnetic field. Apparently, magnetic susceptibility of the specimens aged at 450–550 °C decreases markedly with aging time up to 1000 h, then gradually approaches a steady state value with increasing time. At 475 °C, the reduction in susceptibility is more pronounced. The marked reduction in magnetic susceptibility at the earlier stage of aging is due to the decomposition of ferromagnetic primary ferrite to paramagnetic Cr-rich α’-phase and ferromagnetic Fe-rich α-phase. The magnetic susceptibility of aged specimens does not diminish to zero due to the formation of ferromagnetic Fe-rich α phase. Moreover, primary ferrite that does not fully undergo spinodal decomposition also contributes to the susceptibility. In contrast, magnetic susceptibility of the steel aged at 350 °C remains nearly unchanged, indicating no spinodal decomposition of primary ferrite during aging.

## 3. Corrosion Properties

As recognized, protective oxides or hydroxides can form on the surfaces of transition metals such as Fe, Cr, Ni, Mo and their alloys. The oxide film with a thickness of several nanometers protects underlying metal/alloy from corrosive environment, commonly terming as the passive film. Therefore, annealed DSSs are well protected by the passive films formed on their surfaces. The protectiveness of passive film depends greatly on the Cr, Mo and N contents. In particular, Cr and Mo in the passive film act synergistically in resisting the attack of chloride ions by rehealing damaged film. When secondary phase particles and chromium carbides are formed at the grain boundaries of DSSs, the boundaries adjacent to the precipitates are depleted of Cr and Mo. The Cr/Mo-depleted zone near the grain boundaries is much less corrosion resistant than the surrounding grains. Thus the film locally is less protective and the Cr/Mo depleted zone experiences active dissolution (act as the anode) and corrode upon exposure to corrosive environment, while the surrounding grains remain in the passive state (act as the cathode). The active-passive behavior of metals in aqueous solutions can be determined from a plot of applied potential *versus* current density using a potentiostat. This instrument gives a continuously varying potential to the specimen. As the applied potential is varied, the current is continually recorded. The resulting current density-potential plot is known as the polarization curve. At the passivity domain, the current density of metal dissolution decreases drastically associated with the formation of a stable passive film.

**Figure 13:**
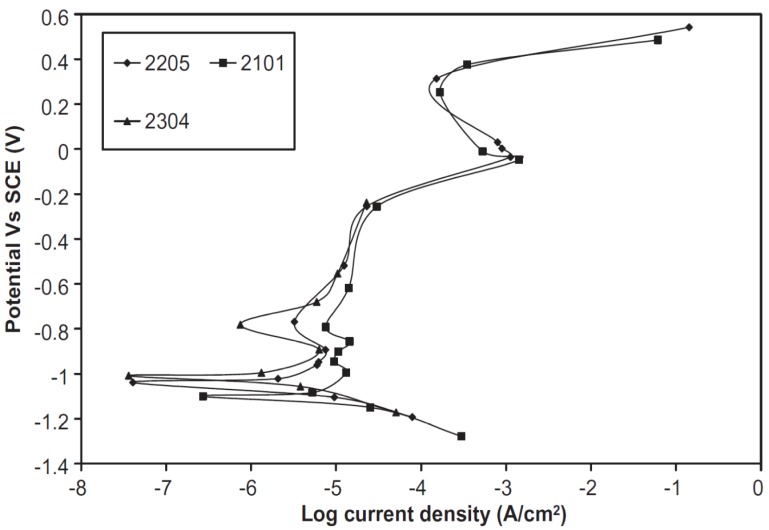
Polarization curves of UNS S32205, S32101 and S32304 steels in 3.75M NaOH solution at 70 °C, reprinted with permission from [47]. Copyright 2011 Elsevier.

**Figure 14:**
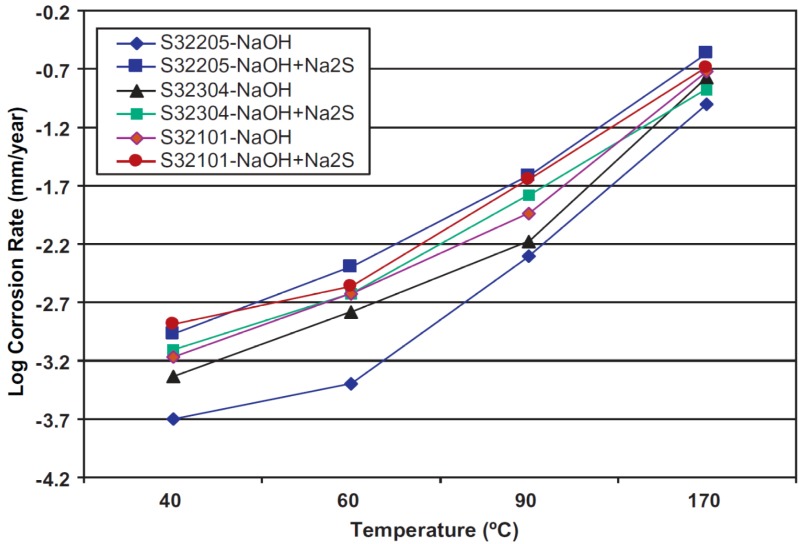
General corrosion rate as a function of temperature determined by immersing UNS S32205, S32101 and S32304 steels in caustic solutions with and without sulfide addition, reprinted with permission from [47]. Copyright 2011 Elsevier.

DSSs exhibit superior corrosion resistance than austenitics in acidic and marine environments. The degree of corrosion protection increases with increasing Cr, Mo and N contents. In addition, DSSs also perform satisfactorily in caustic solutions. Recently, Bhattacharya and Singh investigated corrosion behaviors of the as-received S32205, S32101 and S32304 in both 3.75 M NaOH and 3.75 M NaOH + 0.64 M Na_2_S solutions at 40–170 °C [47]. Sodium sulfide is added because the pulp mill facilities always contain sulfide species. All DSSs exhibit good passivation behavior in the 3.75 M NaOH solution (Figure 13). In this figure, *E*_corr_ of S32205 is located at −1.04 V (SCE). For the potentials more cathodic than *E*_corr_, hydrogen ion reduction reaction takes place as expected. The primary passive region extends from about −1.1 V to −0.3 V with a low current density of ~10^−5^ A/cm^2^. −0.3 to 0 V potential range, the anodic current increases due to the transpassive oxidation of Cr. Transpassive oxidation of a metal is defined as the formation of chemical species in a valence state higher than that in the primary passive film formed on the material. In other words, Cr^III^ species in the passive film of DSS 2205 is further oxidized to Cr^VI^. Above 0 V, secondary passivation occurs followed by the oxygen evolution. Thus, the anodic polarization of S2205 in NaOH solution is rather complex, consisting of primary passivation at lower anodic potential, followed by transpassive oxidation and secondary passivation at high anodic potentials [48]. Furthermore, temperature and Na_2_S species affect general corrosion rates of DSSs in caustic environments (Figure 14). The corrosion rates of these steels in 3.75 M NaOH solution are below 0.2 mm/year even at 170 °C. At temperatures ≤90 °C, the rates are an order of magnitude smaller. In the presence of Na_2_S, the passivation of DSSs in alkaline solution degrades considerably, leading to an increase in the critical current density for passivation and a reduction in the passivation range. Bhattacharya and Singh attributed this to the formation of metal-sulfide compounds in DSSs. Such sulfide compounds are less protective than passive films enriched with Cr-oxide/hydroxide. S32205 is more susceptible to general corrosion than lean DSSs because Mo in the S32205 undergoes active dissolution. Lean S32304 DSS exhibits the lowest corrosion rates in caustic sulfide environmenent due to its lowest Mo content of 0.2 wt%.

### 3.1. Intergranular Corrosion

As mentioned above, the σ-phase formation impairs Charpy impact toughness of DSSs significantly (Figure 7). Moreover, the formation of intermetallic compounds, chromium nitrides and chromium carbides depletes Cr or both the Cr and Mo in DSSs, leading to intergranular corrosion upon exposure to corrosive environments. Accordingly, ASTM A923-03 standard was set up to detect detrimental intermetallic phases in duplex austenitic/ferritic stainless steel that lead to low toughness and poor corrosion resistance [49]. The practice includes method A-sodium hydroxide etch test, method B-Charpy impact test, and method C-ferric chloride corrosion test. Test method A is a screening test for methods B and C. Method C is an immersion weight loss test in ferric chloride solution for 24 h, showing the loss of corrosion resistance due to the depletion of Cr and Mo associated with the precipitation of Cr-rich and Mo-rich phases. From these, it appears that only method C is a quantitative practice for evaluating susceptibility of DSSs to intermetallic compounds. Performing of weight-loss measurements in corrosive media is destructive. Thus, it is necessary to develop other corrosion methods for detecting IGC.

Single loop electrochemical potentiokinetic reactivation (EPR) was originally developed for evaluating the degree of sensitization of austenitic stainless steels [50]. The EPR test is a quantitative method by measuring the amount of charge resulting from dissolution of Cr-depleted regions. In this test, sensitized specimen is first passivated in 0.5 M H_2_SO_4_ + 0.01 M KSCN solution at 0.2 V *vs.* saturated calomel electrode (SCE) for 2 min. This is followed by scanning the potential towards active direction at a rate of 6 V/h, down to the *E*_corr_. The area under the reactivation peak (charge Q) is normalized by the grain boundary area (grain size), reflecting the degree of sensitization (DOS). In contrast, unsensitized stainless steel yields no reactivation peak. Single EPR test requires fine surface finish (1 μm) and grain size determination for detecting DOS [51].

**Figure 15:**
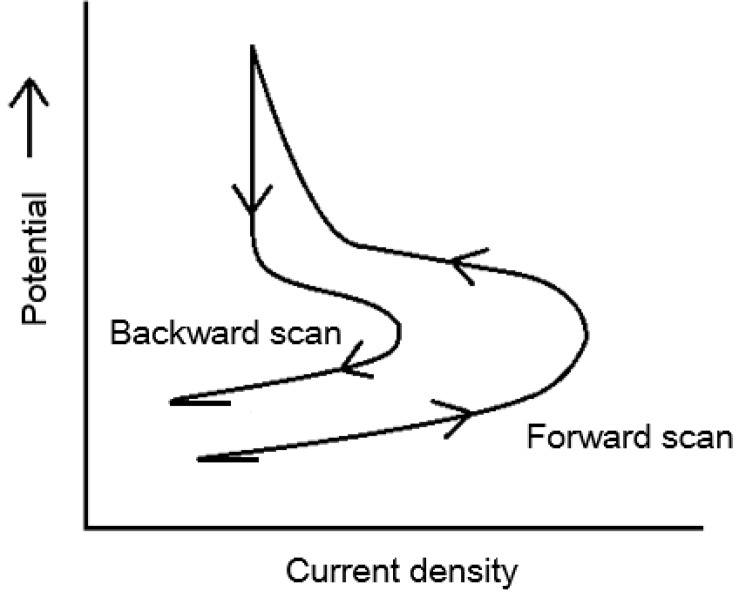
Schematic diagram showing double loop electrochemical potentiokinetic reactivation (EPR) test.

Majidi and Streicher [52] modified the test by polarizing austenitic UNS S30400 in 0.5 M H_2_SO_4_ + 0.01 M KSCN solution from open-circuit potential to the passive region with a constant scan rate. Subsequently, the scan is reversed towards active potential region, *i.e.*, open-circuit potential with the same scan rate. The DOS is defined by the *I*_r_/*I*_a_ × 100, where *I*_a_ is maximum current density in forward scan (activation) and *I*_r_ is the peak current density in reverse scan (reactivation) as shown in Figure 15. On the reverse scan, the passive film formed on Cr-depleted regions during the forward scan degrades considerably, leading to corrosion attack in these areas and generating current peak *I*_r_. This procedure becomes the basis of the ISO 12732 practice [53], and widely known as double loop electrochemical potentiokinetic reactivation (DL-EPR) test. Microstructural factors such as the grain size and surface finish are not accounted for the DL-EPR test. Thus grinding the samples in fine #600 grit SiC paper is commonly adopted. It offers an opportunity for nondestructive measurement of the DOS of stainless steels of different grades with great simplicity [54].

Lopez *et al.* [18] employed DL-EPR test to study the effect of σ-phase formation on the IGC of UNS S31803 aged at 600–900 °C for different times. The test solution was modified to 2 M H_2_SO_4_ + 0.01 M KSCN + 0.5 M NaCl solution at 30 ± 1 °C. KSCN is less effective to de-passivate DSS with better corrosion resistance, thus NaCl acting as a depassivator is added simultaneously. The scan rate used was 1.66 mV/s. They reported that DL-EPR test is effective for detecting DOS resulting from the dissolution of Cr-depleted regions. The DL-EPR results agree closely with those of oxalic acid etch test and corrosion rate measurement (immersion test). Very recently, Ortiz *et al.* [55] also evaluated IGC susceptibility of UNS S31803 in 2 M H_2_SO_4_ + 0.01 M KSCN + 0.5 M NaCl solution at room temperature using DL-EPR method at a scan rate of 1 mV/s. The steel specimens were aged at 700 °C from 1 min to 240 h. The DOS values of all specimens are listed in Table 2. Apparently, the as-received steel and specimens aged for times ≤1 h exhibit very small DOS values, demonstrating no IGC attack. By increasing aging times to 6 h and above, the DOS values increase to about three orders of magnitude higher due to the precipitation of σ-phase.

**Table 2:** Degree of sensitization (DOS) values of as-received UNS S31803 steel and DSS specimens aged at 700 °C for different times, reprinted with permission from [55]. Copyright 2013 Elsevier.

Specimen	Activation Peak Current Density, *I*_a_ (A/cm^2^)	Reactivation Peak Current Density, *I*_r_ (A/cm^2^)	DOS (*I*_r_*/I*_a_) × 100%
As received	0.0163	1.59 × 10^−6^	9.74 × 10^−3^
1 min	0.0126	2.63 × 10^−6^	2.09 × 10^−2^
30 min	0.0177	4.16 × 10^−6^	2.34 × 10^−2^
1 h	0.0066	5.06 × 10^−6^	7.66 × 10^−2^
6 h	0.0240	0.0041	17.40
12 h	0.0251	0.0083	33.15
24 h	0.0472	0.0228	48.37
48 h	0.0579	0.0376	64.91
120 h	0.0830	0.0579	69.69
240 h	0.0753	0.0669	88.83

**Figure 16:**
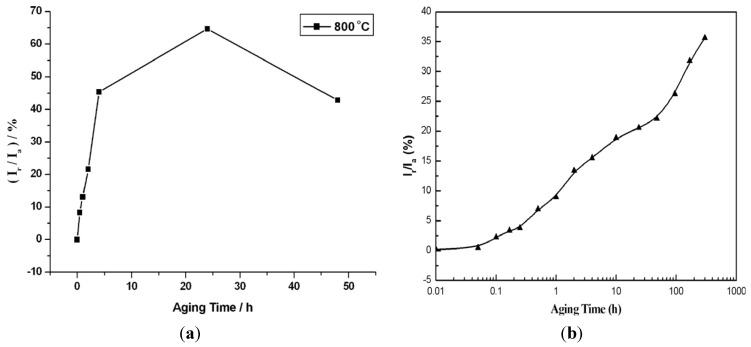
DOS *vs.* aging time of (**a**) UNS S31803 steel treated at 800 °C, reprinted with permission from [57]. Copyright 2010 Elsevier; and (**b**) UNS S32101 steel treated at 700 °C, reprinted with permission from [36]. Copyright 2010 Elsevier.

To improve sensitivity of DL-EPR, HCl depassivator of different concentrations, electrolyte temperatures and scan rates were investigated by some researchers [23,36,56,57,58]. Hong *et al.* [23] reported that the optimal conditions for evaluating DOS of S32750 in a 2 M H_2_SO_4_ solution can be obtained by using 1.5 M HCl, a scan rate of 1.5 mV/s and a solution temperature of 30 °C. Gong *et al.* [57] reported that an electrolyte of 2 M H_2_SO_4_ + 1 M HCl at 30 °C and a scan rate of 1.66 mV/s give optimal DL-EPR responses for aged UNS S31803. Figure 16a shows the DOS *vs.* aging time for S31803 determined under these conditions. The DOS reaches an apparent maximum value of 64.8% by aging at 800 °C for 24 h, and then decreases to 42.85% as the aging time increases to 48 h. The decrease in DOS value is attributed to the self-healing of Cr-depleted zones associated with the diffusion of Cr and Mo from the matrix to depleted regions. Such self-healing behavior also occurs in S32750 [23]. For lean UNS S32101 DSS, an electrolyte of 33% H_2_SO_4_ + 0.1% HCl at 20 °C and a scan rate of 2.5 mV/s give the best reproducibility [36]. The DOS values *vs.* aging time are shown in Figure 16b. Apparently, the DOS values increase markedly with increasing aging time. The initial small increase in DOS value is caused by the depletion of Cr associated with the Cr_2_N precipitation as mentioned previously. The IGC becomes more serious once the σ-phase begins to nucleate in this steel (Figure 8), producing large DOS value of 31.5% as expected. There is no self-healing in lean DSS after aging up to 300 h. Amadou *et al.* [56] studied the applicability of the DL-EPR method for detecting intergranular corrosion susceptibility of UNS S31260 and S31803 steels aged at 500 °C to 900 °C for different periods ranging from 6 min to 120 h. The electrolyte and scan rate used were 33% H_2_SO_4_ + 0.3% HCl (room temperature) and 2.5 mV/s. A wide range of aging temperatures was selected in order to induce precipitation of Cr_23_C_6_ carbides (550–650 °C) and intermetallic phases (650–850 °C). DOS was also defined from the ratio of reactivation charge (*Q*_r_) and activation charge (*Q*_a_) given by the relation *DOS* = *Q*_r_/*Q*_a_. Solution annealed steel exhibits DOS value with *Q*_r_/*Q*_a_ < 1, or I_r_/I_a_ < 1. In contrast, sensitized steel displays the *Q*_r_/*Q*_a_ ≥ 1, or *I*_r_/*I*_a_ ≥ 1 behavior. They demonstrated that the DL-EPR technique is very effective to detect IGC caused by the Cr-depletion zone due to the precipitation of Cr_23_C_6_ carbides, χ and σ phases in DSSs.

**Figure 17:**
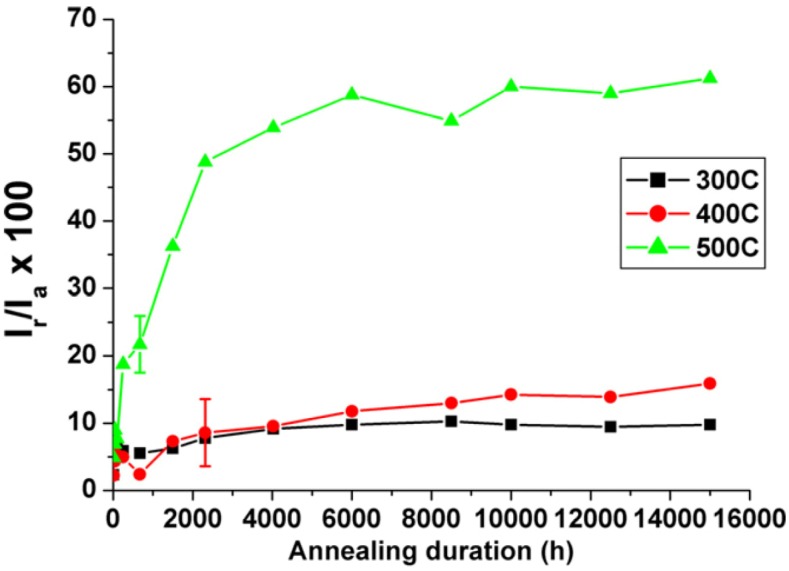
(*I*_r_/*I*_a_ × 100) value *vs.* aging time plots of 7MoPLUS steel aged at 300–500 °C, reprinted with permission from [26]. Copyright 2012 Elsevier.

It is worth noting that DL-EPR test can also be used to characterize the Cr depletion in DSSs due to the formation of Cr-rich α’-precipitates during the spinodal decomposition [26]. Figure 17 shows the plots of (*I*_r_/*I*_a_ × 100) *vs.* aging time for the 7MoPLUS steel aged at 300–500 °C for extended periods. At low temperatures of 300 and 400 °C, the (*I*_r_/*I*_a_ × 100) values of aged specimens remain relatively low even after prolonged aging for 15,000 h. However, the (*I*_r_/*I*_a_ × 100) value increases significantly at 500 °C due to the formation of Cr-depletion zone next to the Cr-rich α’-phase.

From these results, it appears that DL-EPR can be used to detect the Cr depletion zone resulting from the precipitation of Cr_23_C_6_ carbides, intermetallic compounds and Cr-rich α’-precipitates. Therefore, the reproducibility of the test is considered of technological importance. The sensitivity of DL-EPR depends greatly on the operating condition (e.g., scan rate), electrolyte temperature and pH, depassivator content, *etc.* At present, there is no general agreement among researchers concerning the concentrations of electrolyte and depassivator, the scan rate and electrolyte temperature for this test [23,36,55,56,57,58]. The optimal conditions for achieving good reproducibility are still under investigation. Amadou *et al.* [56] determined the DOS from the ratio of reactivation charge and activation charge during the DL-EPR test, but the DOS value was not normalized by the grain size (grain boundary area) of duplex steels as the DOS in austenitic stainless steels. Therefore, careful consideration should be given to the issues of microstructural and electrolyte parameters such that the DL-EPR test can yield data with high reproducibility.

### 3.2. Localized Corrosion

It is well recognized that crevice and pitting corrosion are caused by a breakdown of the passive films of stainless steels exposed to chloride containing environments. Chloride ions can induce failure of the passive film formed on the inclusions (e.g., MnS) and secondary phases, leading to anodic dissolution of underlying metal locally. The adjacent metal surfaces act as the cathode for oxygen reduction process. Rapid dissolution at localized region initiates the pit formation. An excess of metallic ions with positive charge is accumulated in this region, resulting in the migration of chloride ions from the solution to maintain electroneutrality. Consequently, a high concentration of MCl within the pit undergoes hydrolysis, producing low pH due to the formation of hydrochloric acid. This increases the local dissolution rate causing more chloride ions to migrate into the pit. The process is a self-propagating or autocatalytic mechanism of pit growth [59]. Moreover, pitting can lead to other causes of failure such as stress corrosion. The simultaneous presence of tensile stress and corrosive environment can breakdown the passive film even more readily, forming fine cracks that fracture in a brittle manner. These cracks propagate across the grains in either transgranular or intergranular mode. This phenomenon is referred to as the “stress corrosion cracking” (SCC). Residual or thermal stresses resulting from the fabrication, improper heat treatment and welding process of DSSs form the basis for tensile loads. Moreover, the presence of intermetallic phases at the α/α and α/γ grain boundaries facilities the cracks to propagate along those boundaries.

#### 3.2.1. Pitting Corrosion

The pitting resistance of DSSs in marine environment depends mainly on their Cr, Mo and N contents. Thus, the pitting corrosion resistance is correlated with the chemical compositions of DSSs, and can be expressed in term of an empirical pitting resistance equivalent number (PREN) given by [12]:

PREN = wt% Cr + 3.3 wt% Mo + *x*wt% N (2)

where the *x* value ranges from 16 to 30. Larger PREN value gives rise to higher pitting resistance. There is no universal equation for evaluating PREN of DSSs. A value of *x* = 16 is typically adopted in industrial sector [16], while *x* = 20 is commonly used by the researchers [60,61,62]. For *x* = 16, the PREN values of standard 2205 grade, 25% Cr, superduplex and hyperduplex steels are ~ 35, 35–40, 40–50 and ≥50, respectively [16]. Equation (5) only takes into account the beneficial effect of Cr, Mo and N on the pitting corrosion resistance of DSSs. However, other elements such as Mn, S and P that exhibit deleterious effect on the pitting resistance are ignored. Partitioning of Cr and Mo in ferritic phase, and of Ni and N in austenitic phase can affect the PREN values of both phases. Furthermore, the precipitation of secondary phase particles can cause compositional changes in the α- and γ-phases, resulting in selective pitting corrosion of a weak phase [22].

**Figure 18:**
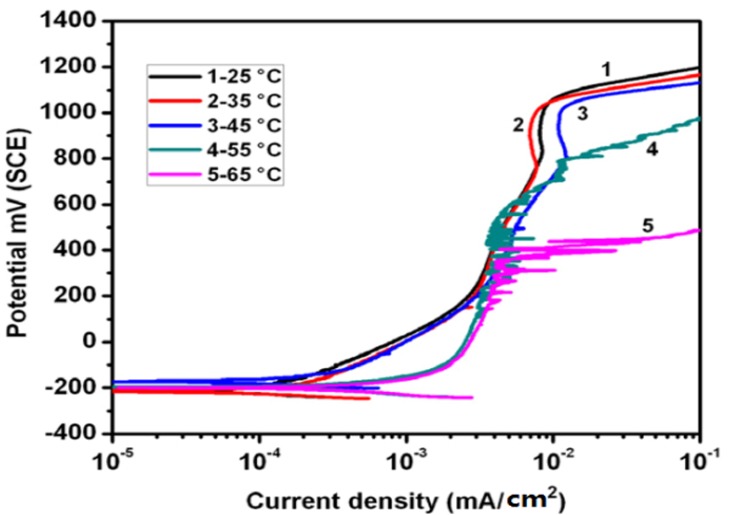
Polarization curves of 2205 DSS exposed in 0.1 M NaCl solution at different temperatures, reprinted with permission from [63]. Copyright 2012 Elsevier.

In addition to intrinsic material properties, environmental parameters such as chloride concentration, pH and temperature of the solutions also affect the pitting corrosion resistance of DSSs. Figure 18 shows the effect of temperature on polarization behavior of solution-annealed DSS 2205 in 0.1 M NaCl solution. Apparently, the current density of DSS in passivation region increases with increasing solution temperatures [63]. Moreover, the pitting potential of DSS shifts to less noble value as the solution temperature increases. Pitting potential in the polarization curve is characterized by the dramatic increase in current density at a critical potential. This potential is taken as a measure of resistance to pitting corrosion. The pitting potential of DSS 2205 in 0.1 M NaCl solution at 25–45 °C is 1050 mV, but shifts to more active value of 400 mV as the solution temperature increases to 65 °C. It is noted that the scans do not show a true pitting potential at 25–45 °C since the current increases into transpassivity at 1050 mV.

**Figure 19:**
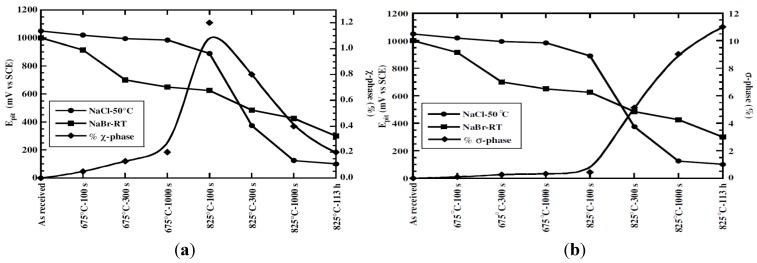
Variation of pitting potential of S32760 exposed in halide solutions with (**a**) χ-phase and (**b**) σ-phase volume fraction, reprinted with permission from [64]. Copyright 2006 Elsevier.

Domínguez-Aguilar and Newman studied the deleterious effects of secondary phase formation on the pitting behavior of UNS S32760 containing 24.97 wt% Cr and 3.58 wt% Mo exposed in 0.85 M halide solutions (NaBr, NaCl) at different temperatures (20–50 °C) [64]. The steel was aged at 67–825 °C for different periods to induce the formation of χ and σ phases. As mentioned above, Mo promotes the formation of χ- and σ-phases in DSSs in which the χ-phase nucleates in the early stage of aging. In both halide solutions the presence of intermetallics leads to pitting corrosion. The Cr/Mo depleted zones are preferred dissolution sites in halide solutions. The passive films formed on these zones are less protective because they are depleted of Cr and Mo. The bromide solution is more effective to detect mild solute depletion at room temperature, while chloride solution at room temperature is ineffective and must be heated to 50 °C. Figure 19a shows the effects of aging temperature and time as well as χ-phase volume content on the pitting potential of S32760 exposed in halide solutions. The pitting potential of this steel immersed in 0.85 M NaCl solution (50 °C) and 0.85 M NaBr solution (room temperature) decreases with increasing χ-phase content as expected. The correlation between pitting potential and σ-phase volume fraction is shown in Figure 19b, which shows a shift of pitting potential to more active region as the σ-phase content increases.

As recognized, pitting potential determined from anodic polarization tests depends greatly on the scan rate and fine crevice formed at the sample/resin interface. For the sample mounted in a resin, the crevice effect lowers its pitting potential considerably. In this respect, critical pitting temperature (CPT) is more appropriate to describe the susceptibility of stainless alloys to pitting corrosion [65]. From the ASTM G150 test, CPT values of stainless steels can be determined using potentiostatic polarization [66]. In this test, an anodic potential of 700 mV *vs.* SCE is applied to the specimen immersed in 1 M NaCl solution. The current density is recorded while the solution temperature is continuously increased at a rate of 1 °C/min until stable pitting occurred. CPT is defined as the temperature when the sample current density reaches 100 μA/cm^2^.

**Figure 20:**
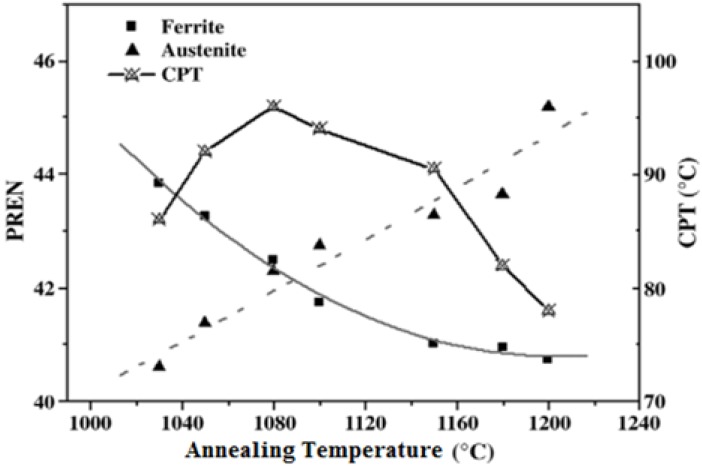
Variations of α- and γ-phase pitting resistance equivalent number (PREN) of UNS S32750 and its critical pitting temperature (CPT) in 1 M NaCl solution with annealing temperature, reprinted with permission from [33]. Copyright 2009 Elsevier.

**Figure 21:**
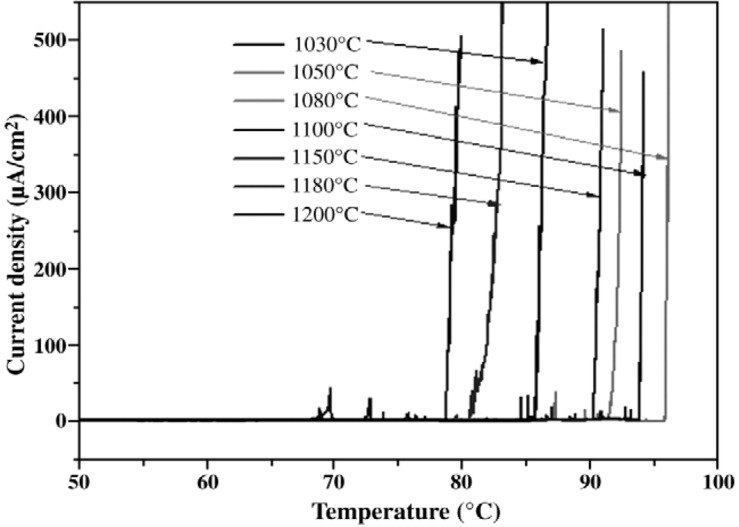
Potentiostatic CPT measurements of UNS S32750 in 1 M NaCl solution under a potential of 750 mV(SCE), reprinted with permission from [33]. Copyright 2009 Elsevier.

Li *et al.* [33,60] studied the effect of microstructural evolution on the CPT behavior of lean S32304 and superduplex S32750 steels by annealing at 1000–1200 °C for 1–2 h followed by water quenching. Annealing at high temperatures disturbs the balance of α- and γ-phases in these steels. From the results of image analysis, the ferrite volume fraction increases while the austenite fraction decreases with increasing temperature. Energy dispersive X-ray measurements reveal that the Cr and Mo contents in the α-phase decrease while N content in the γ-phase increases with increasing temperature. Accordingly, PREN value determined with *x* = 20 for the α-phase decreases, while that for the γ-phase rises by increasing the temperature (Figure 20). At a cross-over temperature of 1080 °C, PREN values of both phases are almost the same, demonstrating that the α- and γ-phases have equal pitting resistance. Consequently, pits are nucleated at α/γ boundaries. Below 1080 °C, PREN value of γ-phase is smaller than that of α-phase, implying that α-phase is more corrosion resistant. Therefore, pits are preferentially nucleated in the γ-grains. Above 1080 °C, PREN value of α-phase is smaller than that of γ-phase, thus pits are nucleated in the α-phase accordingly. Figure 21 shows the application of potentiostatic polarization method for determining CPT of annealed UNS S32750 in 1 M NaCl solution. CPT values are determined from potentiostatic measurements by applying an anodic potential of 750 mV (SCE) to the specimen and continuously increasing solution temperature at a rate of 1 °C/min until stable pitting occurred. CPT is defined as the temperature at which the current density reaches 100 μA/cm^2^. Treating S32750 at 1080 °C produces the highest CPT of 96 °C, *i.e.*, the steel exhibits the best pitting resistance at this temperature. The CPT *vs.* annealing temperature of S32750 is also plotted in Figure 20. The CPT value generally follows rise/fall PREN trend of less corrosion resistant phase. In other words, CPT first increases with annealing temperature up to 1080 °C and follows rising trend of austenitic PREN, then decreases with annealing temperature and follows falling trend of ferritic PREN. Table 3 summarizes the effect of annealing temperature on pitting susceptibility of S32750 and S32304 steels. Lean S32304 exhibits low CPT values in 1 M NaCl solution as expected due to its extremely low Mo (0.3 wt%) and lower Cr (22.9 wt%).

**Table 3:** Effect of annealing temperature on pitting behavior of S32750 and S32304 steels [33,60].

Material	Annealing Temperature, °C and Time	α-Phase Content, vol%	γ-Phase Content, vol%	CPT (°C), 1 M NaCl	Pitting Phase
S32750	1050, 2 h	47	53	92	γ
	1080, 2 h	49	51	96	α/γ boundary
	1150, 2 h	55	45	90	α
	1200, 2 h	62	38	78	α
S32304	1030, 1 h	47	53	58	γ
	1080, 2 h	51	49	67.8	α/γ boundary
	1150, 1 h	58	42	56	α
	1200, 1 h	63	37	52	α

**Figure 22:**
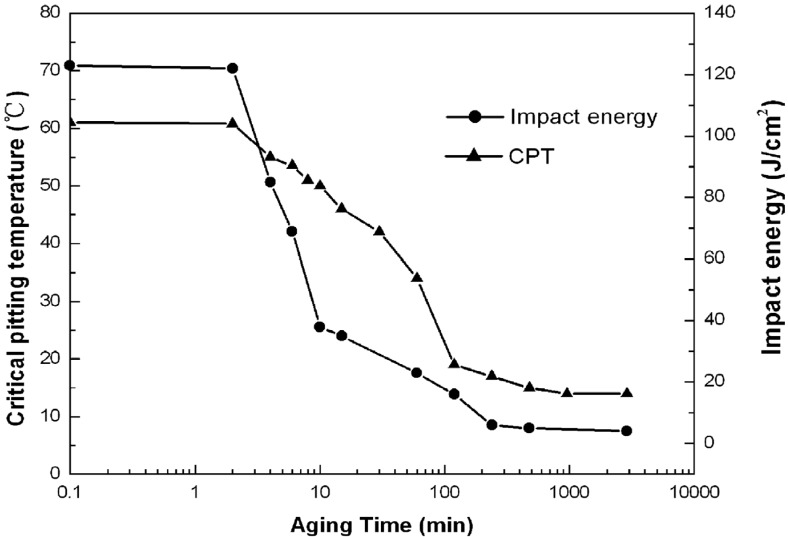
Critical pitting temperature and impact energy *vs.* aging time for UNS S31803, reprinted with permission from [62]. Copyright 2009 Elsevier.

**Figure 23:**
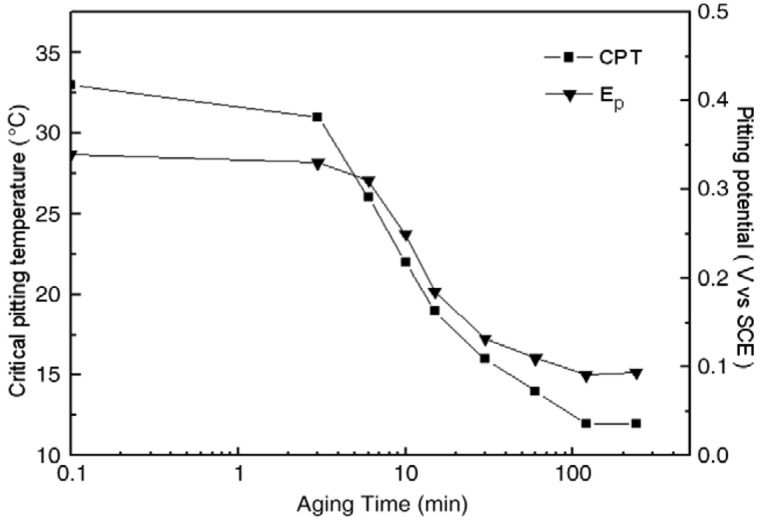
CPT and pitting potential (*E*_p_) of UNS S32101 in 1 M NaCl solution *vs.* aging time, reprinted with permission from [35]. Copyright 2009 Elsevier.

**Figure 24:**
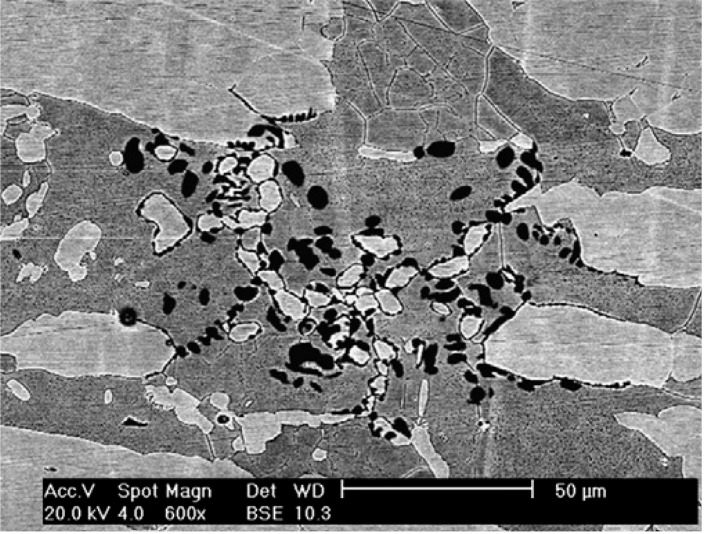
SEM image showing formation of pits in UNS S32101 steel aged at 700 °C for 240 min upon exposure to 1 M NaCl solution. Light phase is austenite and dark phase is ferrite, reprinted with permission from [35]. Copyright 2009 Elsevier.

The effect of σ-phase formation on the CPT behavior of DSSs is now considered. Aging DSSs at high temperatures generally leads to a decrease in their CPT values [21,22,62]. Figure 22 shows the CPT *vs.* aging time for S31803 treated at 850 °C in deaerated 1 M NaCl solution. The CPT value of solution-annealed steel is 61 °C and drops considerably with aging time, and reaches a steady value of ~14 °C after aging for 480 min (8 h). For comparison, the impact strength *vs.* aging time of S31803 is also plotted in this figure. The impact strength of solution-annealed specimen drops sharply with increasing aging time since the amount of σ-phase increases with time. For lean UNS S32101 DSS contains very low Mo content, its pitting potential (at 30 °C) and CPT in 1 M NaCl solution also decrease with aging time (Figure 23) [35]. These can be attributed to the formation of Cr_2_N precipitates at the α/α and α/γ grain boundaries during an earlier stage of aging at 700 °C as mentioned previously. Pits nucleate mainly at the Cr-depletion region around the Cr_2_N precipitates, and then grow into ferritic grains (Figure 24).

**Figure 25:**
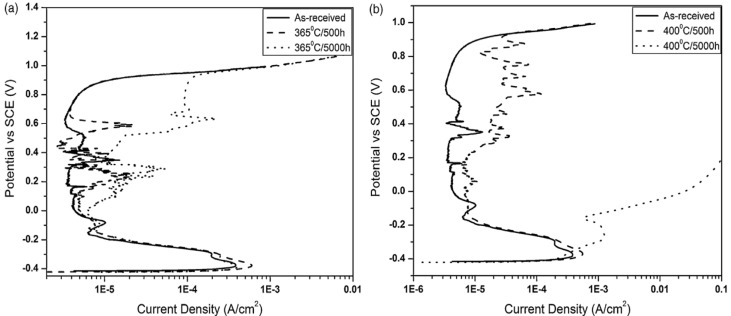
Anodic polarization curves of DSS2205 in 1 M HCl solution aged at (**a**) 365 °C and (**b**) 400 °C for different times, reprinted with permission from [39]. Copyright 2010 Elsevier.

**Figure 26:**
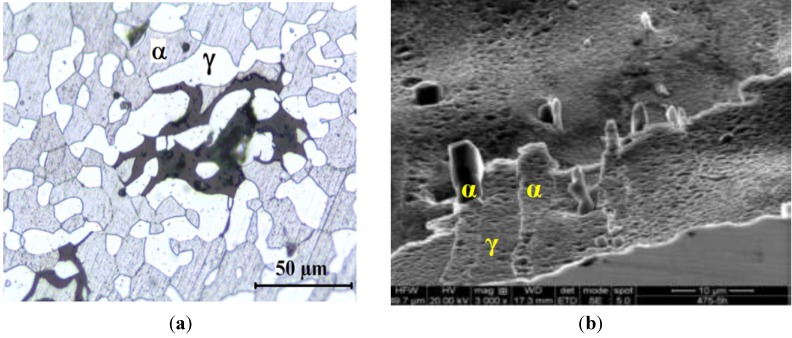
(**a**) Optical micrograph of DSS 2205 aged at 400 °C for 5000 h after polarization testing in 1 M HCl solution, reprinted with permission from [39]. Copyright 2010 Elsevier; (**b**) SEM image of DSS aged at 475 °C for 5 h after CPT testing, reprinted with permission from [37]. Copyright 2013 Institute of Corrosion, UK.

It is worth noting that spinodal decomposed phases in DSSs also impair their pitting resistance. Figure 25a,b show anodic polarization curves of the as-received and aged DSS 2205 specimens in 1 M HCl solution. The Ecorr of the as-received steel is located at −0.39 V (SCE). By increasing potential from the Ecorr anodically, passivation occurs and extends from about −0.2 V to 0.85 V (SCE) with a passive current density of 6 × 10^−6^ A/cm^2^. Beyond 0.85V (SCE), pitting occurs due to a dramatic increase in the current density. The passive range and pitting potential of aged samples at 365 °C for different periods and the sample at 400 °C for 500 h remains almost unchanged. However, the passivation behavior is largely disturbed by aging at 365 °C for 5000 h as well as 400 °C for 500 h and 50,000 h. The passive current density of sample aged at 400 °C for 500 h increases to ~2 × 10^−5^ A/cm^2^ (Figure 25b). The pitting potential drops markedly to −0.16 V (SCE) after prolonged aging at 400 °C for 5000 h. This is accompanied by a marked reduction in the passivation range. This is caused by the formation of α’-phase, leading to serious pitting attack in the ferrite phase (Figure 26a). Ornek *et al.* [37] reported that spinodal decomposition is benefical for the corrosion resistance of DSS 2205 aged at 475 °C for up to 10 h because of a better passivation behavior. The CPT value in 3 wt% NaCl solution for the sample aged for 5 h is 50 °C compared with annealed steel of 40 °C. Figure 26b shows a lesser corrosion attack in the α-phase of this aged sample after CPT testing. Prolonged aging for 255 h causes a sharp drop in the CPT value to 30 °C owing to the formation of elemental depletion zone [37].

#### 3.2.2. Stress Corrosion Cracking

DSSs generally exhibit higher resistance to SCC in chloride containing solutions than austenitic stainless steels. Therefore, DSSs can replace austenitic grades in the chemical process industries in applications with a high risk of SCC. The resistance of DSSs to SCC is markedly reduced by high temperature, low pH and high applied stress [4]. Constant deformation U-bend, constant load dead-weight, slow strain rate test (SSRT) and fracture mechanics techniques are commonly used for testing SCC susceptibility in aggressive solutions [5,67,68,69,70,71,72,73,74]. In particular, SSRT method (ASTM 129) is performed under tensile loading at low strain rates (e.g., 10^−6^–10^−7^/s) [75]. At a critical strain rate, the samples display a ductility minimum, showing the occurrence of brittle failure. The emergence of dislocations at the surface due to their motion along the slip planes disrupts the passive film, resulting in anodic dissolution of metal at highly localized areas. Generally, solution compositions affect the crack initiation process in DSSs. The α-phase is more susceptible to SCC attack in hot chloride solutions [70], while the γ-phase is selectively attacked in hot alkaline sulfide solutions [71,73]. Besides, microstructural anisotropy of hot rolled DSSs along the rolling longitudinal and transverse longitudinal directions influences the nucleation and growth of fine cracks. This anisotropy affects the SCC susceptibility by favoring crack initiation in the γ-phase and at phase interfaces [73].

**Figure 27:**
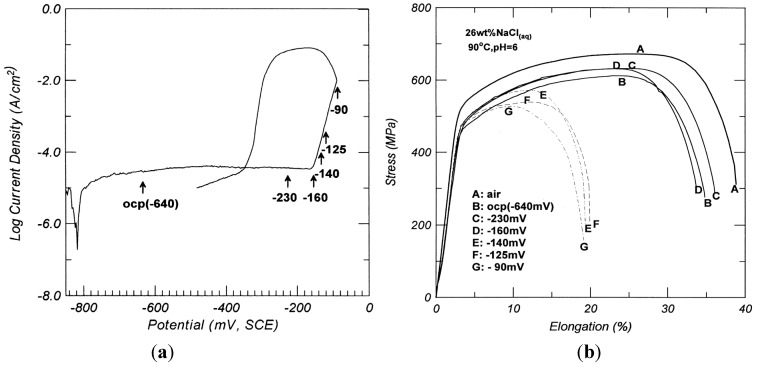
(**a**) Cyclic polarization curve of annealed DSS 2205 in 26 wt% NaCl solution at 90 °C; (**b**) Effect of applied potentials on SCC susceptibility of DSS 2205 in 26 wt% NaCl solution at 90 °C at 4.1 × 10^−6^/s, reprinted with permission from [69]. Copyright 2000 Elsevier.

Tsai and Chen reported that annealed DSS 2205 is immune to SCC in a 26 wt% NaCl solution at open-circuit potential (OCP) from 25 to 90 °C, but susceptible to SCC at high anodic potentials [69]. The pitting potential of DSS 2205 in this solution at 90 °C is −160 mV (SCE) (Figure 27a). By applying potentials more noble than this value, the tensile elongation of annealed DSS 2205 reduces markedly (Figure 27b). Thus corrosion pits assist fine crack nucleation, causing selective dissolution of the α-phase. In another study, [70], it was found that metal cations affect the activities of Cl^−^ and H^+^ ions for initiating SCC with the ability in the following trend: Mg^2+^ > Ca^2+^> Fe^2+^ > Na^+^ > Li^+^ [67,68].

**Figure 28:**
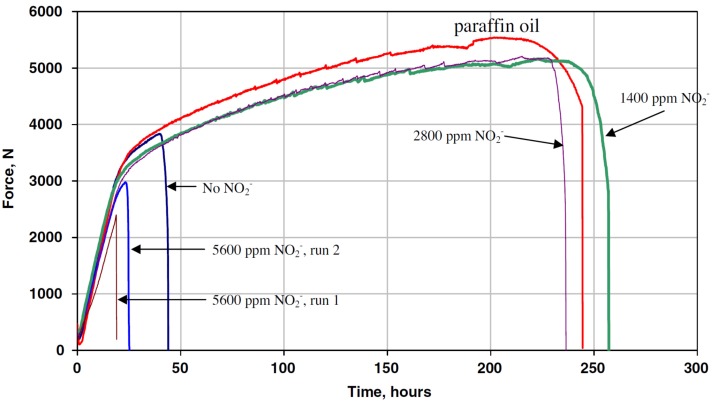
Force *vs.* time slow strain rate test (SSRT) curves for SAF 2507 in 30 wt% MgCl_2_ solution at 180 °C at a strain rate of 3.7 × 10^−7^/s with different NO_2_^−^ concentrations, reprinted with permission from [72]. Copyright 2010 Elsevier.

Recently, Singh-Raman and Siew studied SCC behavior of superduplex SAF 2507 and its suppression in a 30 wt% MgCl_2_ solution at 180 °C using SSRT method under open-circuit potential condition [72]. This steel experienced intergranular cracking at a strain rate of 3.7 × 10^−7^/s. To inhibit SCC, NaNO_2_ inhibitor of different concentrations was added to MgCl_2_ solution. They reported that the additions of low concentrations of nitrite, *i.e.*, 1400 and 2800 ppm were very effective to suppress SCC due to the NO_2_^−^ ions inhibit pitting of SAF2507 in the MgCl_2_ solution (Figure 28). Thus pitting assisting crack nucleation was suppressed accordingly. However, addition of 5600 ppm NO_2_^−^ led to the SCC susceptibility, possibly resulting from instability of the passive film at a crack tip.

**Figure 29:**
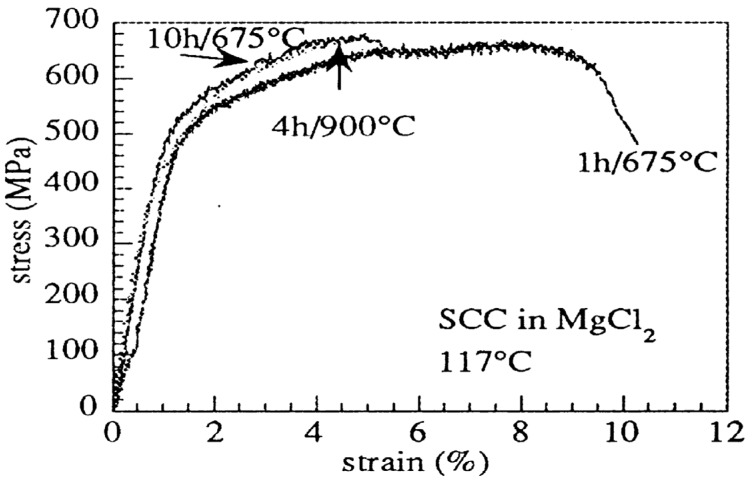
SSRT curves for aged UNS 31803 steel specimens in boiling 30 wt% MgCl_2_ solution at 117 °C, reprinted with permission from [18]. Copyright 1999 Elsevier.

The σ-phase formed in DSSs is detrimental to their resistance against SCC. Figure 29 shows the SSRT results for aged UNS S31803 in boiling 30 wt% MgCl_2_ solution at 117 °C (pH = 5). The steel specimens aged at 675 °C for 10 h and at 900 °C for 4 h are particular vulnerable to SCC as revealed by very low tensile elongation of ~5%. More recently, Saithala *et al.* [74] annealed UNS S32750 superduplex at 1080 °C, followed by aging at 850 °C from 45 s up to 12 min. They reported that low levels of σ-phase (less than 2 vol%) have little effect on the mechanical properties of SDSS exposed to a simulated oil field brine containing carbon dioxide/hydrogen sulfide. At higher levels of σ-phase (>2 vol%), this steel suffers severe loss of ductility during slow rate tensile straining. The SCC mechanism involves failure of σ-phase as a result of brittle fracture followed by pitting-assisted anodic dissolution of the σ- and α-phases. Ubhi *et al.* [76] used EBSD for identifying χ- and σ-phases in aged S32760, and then performed SSRT of aged steel in chloride solutions at 130 °C. They reported that the χ- and σ-phases are detrimental to the resistance of S32760 against SCC. Preferential attack occurred at the α-α and α-γ boundaries due to the formation of σ-phase in these regions.

## 4. Weldment Failure

DSSs find widespread applications in industrial sectors and welding is an important process for fabricating stainless steel structures for engineering applications. DSSs generally have good weldability, but the melting and solidification processes associated with welding destroy favorable duplex microstructure. In addition, welding often causes many defects in the fusion zone (FZ) and heat- affected zone (HAZ) of steel structures [77]. The heat generated during welding process is transferred from the FZ to HAZ. Therefore, FZ and HAZ are the weak points; hence failures of welded components often occur in these regions. The ferrite–austenite phase ratio of DSSs must be maintained close to 50:50 for achieving desired mechanical properties and corrosion resistance. Welding processes tend to upset this phase ratio balance because of slow/fast cooling involved in thermal cycles. The heat input during welding controls the cooling rate and the extent of δ-ferrite → γ transformation [78]. During welding, dissolution of primary austenite occurs initially, followed by grain growth in the δ-ferrite and eventually reformation of austenite during cooling. Austenite tends to nucleate at the grain boundaries, but it can also precipitate in the grain interiors at slow cooling rates [79].

In general, a high heat input or slow cooling rate favors reformation of austenite during cooling. However, intermetallic compounds can form easily in the FZ and HAZ under a slow cooling rate, especially for highly alloyed superduplex and hyperduplex grades [16,80,81]. Consequently, the toughness and corrosion resistance of welded DSSs decrease significantly. On the contrary, a low heat input or fast cooling rate produces an excess of ferrite phase and Cr_2_N particles. The solubility of nitrogen in ferrite is quite low, resulting in supersaturation of nitrogen in ferrite and the precipitation of Cr_2_N upon rapid cooling from high temperatures [82]. The austenite content in the weld zone can be increased by increasing Ni content of the filler materials and/or the use of nitrogen as a shielding gas, and post-welding heat treatment (PWHT) [83]. The microstructures, mechanical properties and corrosion behaviors of welded DSSs depend greatly on the steel compositions and welding techniques employed. Several techniques are typically used for welding DSSs including tungsten inert gas (TIG) welding, gas metal arc welding, plasma arc welding, friction welding, electron beam welding, laser welding, *etc.* Each technique has its own advantages and limitations [1,2,3,4,16,84,85,86]. Apparently, welding can reduce the corrosion resistance of DSSs by changing the steel composition and microstructure in the FZ. The intermetallic phases and Cr-nitrides induced in weld metals of DSSs can lead to poor corrosion resistance upon exposure in aggressive environments [5,87,88]. In this section, the deleterious effects of these phases on the corrosion behavior of welded DSSs are briefly discussed.

**Figure 30:**
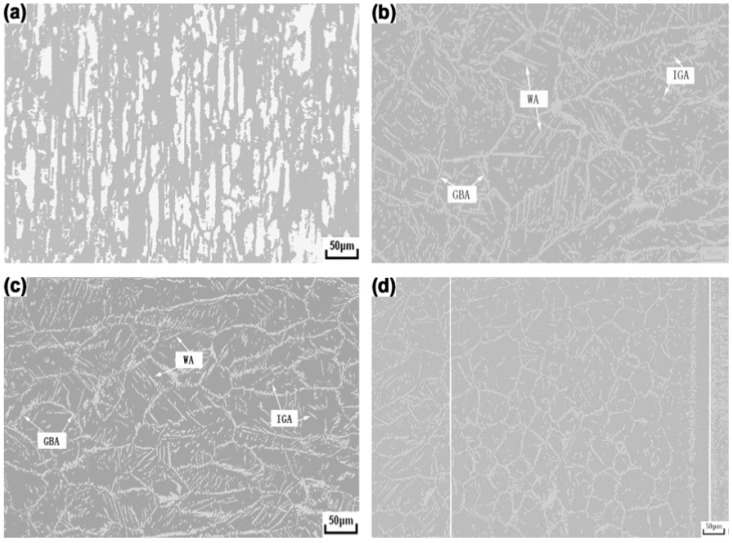
Optical micrographs of (**a**) as-received DSS 2205; (**b**) weld metal near fusion line; (**c**) center of weld metal and (**d**) heat- affected zone (HAZ) (located between two white lines), reprinted with permission from [87]. Copyright 2012 Elsevier.

**Figure 31:**
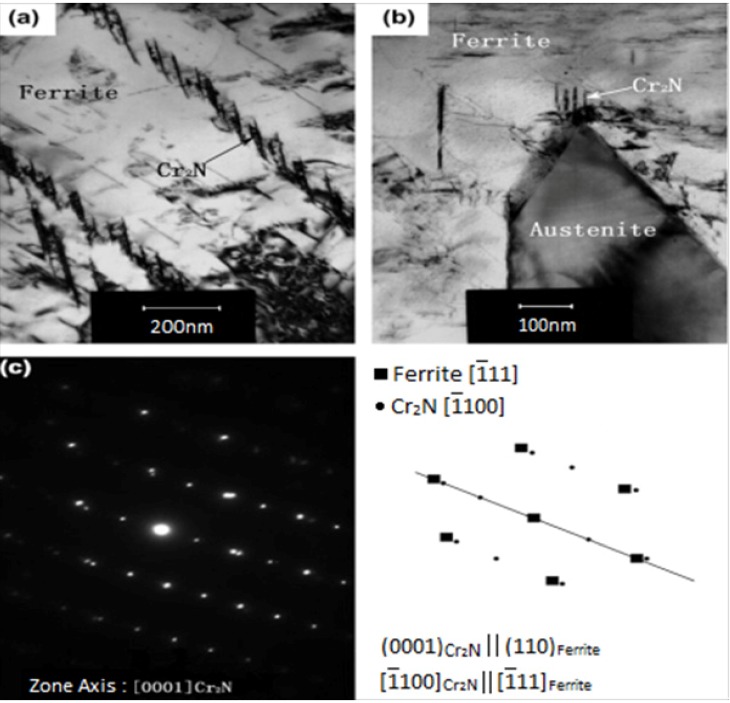
TEM micrographs showing precipitation of Cr_2_N rods at (**a**) fusion zone and (**b**) α/γ boundaries; (**c**) SAED pattern and (**d**) index diagram, reprinted with permission from [87]. Copyright 2012 Elsevier.

Figure 30a–d show optical micrographs of DSS 2205 welded by the TIG welding process under an argon shielding gas. Three types of austenite are formed in the weld metal during fast solidification: (1) austenite nucleated at the prior ferrite grain boundaries (GBA); (2) Widmänstten-type austenite (WA) of plate-like feature nucleated from the grain boundaries; and (3) intragranular austenite precipitates in ferritic grains (IGA). The α-phase content of base alloy, weld metal and HAZ determined from image analysis is 49, 77.5 and 75 vol%, respectively. Figure 31a,b is the TEM micrographs showing the formation of rod-like Cr_2_N at the α/γ boundaries of fusion zone. These Cr_2_N rods grow towards into the ferrite. The SAED pattern and corresponding index diagram are shown in Figure 31c,d, respectively. The formation of Cr_2_N depletes Cr content in the α-phase. The high ferrite content together with the formation of Cr_2_N rods degrade pitting resistance of the weld metal considerably. In this regard, PWHT can be used to restore a favorable balance of α/γ phase ratio. An optimal PWHT is found to be 1080 °C, reducing the α-content in the weld metal sharply from 77.5 to 53.57 vol%. Consequently, the CPT of the fusion zone immersed in 1 M NaCl solution is 58 °C, *i.e.*, close to that of base alloy with a value of 59 °C. Alternatively, N_2_ shielding gas can increase the γ- phase fraction but decrease α-phase and Cr_2_N contents in the weld metal of DSSs. Thus the CPT increases while the corrosion rate decreases with increasing N content in the weld metal [89].

**Figure 32:**
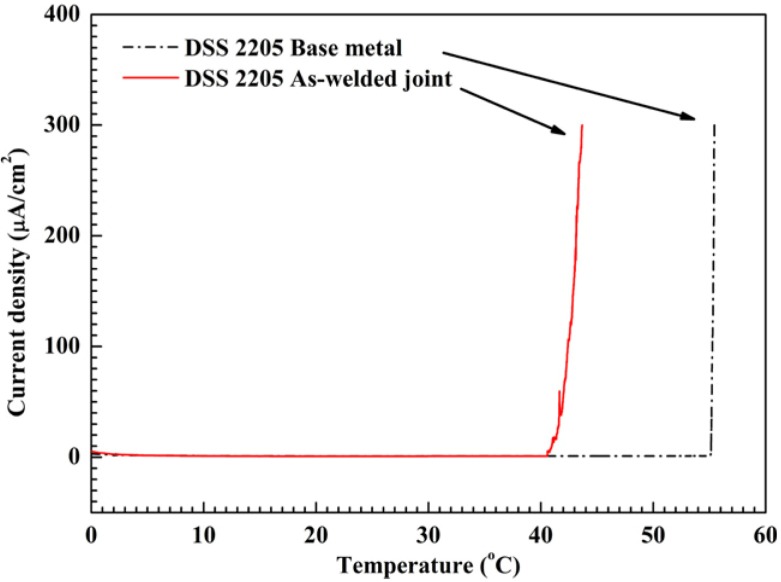
CPT curves of DSS 2205 base metal and laser welded fusion zone, reprinted with permission from [90]. Copyright 2012 Elsevier.

**Figure 33:**
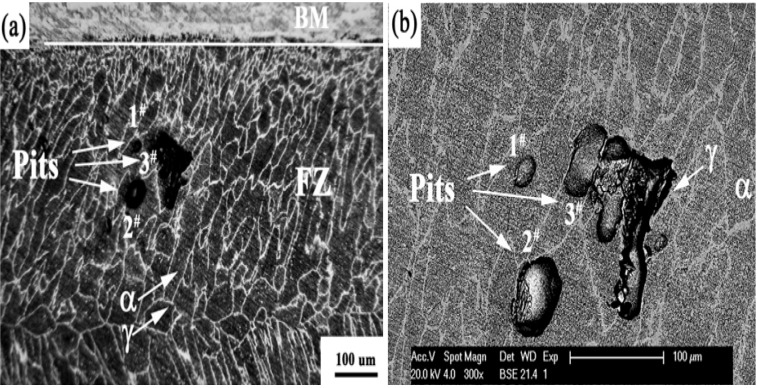
(**a**) Optical and (**b**) SEM images of fusion zone of laser welded DSS2205 steel. Austenite is light and ferrite is dark in (**a**). Pit 1# is located in ferritic grain and pit 2# at the α/γ boundary; large pit 3# spanned across α/γ domains with severely attacked α-grains (BM: base metal, FZ: fusion zone), reprinted with permission from [90]. Copyright 2012 Elsevier.

Laser beams are coherent and intense, thus capable of attaining fast surface melting followed by rapid solidification. These unique features render the weldments with very fine FZ and HAZ. Recently, Yang *et al.* [90] studied the microstructure and corrosion behavior of laser welded UNS S31803 steel. The as-welded joint shows poor pitting resistance due to the high volume fraction of ferrite (92%) and the precipitation of Cr_2_N in the α-phase of fusion zone. CPT measurements in 1 M NaCl solution revealed that the Cr_2_N precipitates in FZ are detrimental to the pitting corrosion resistance. The CPT of base metal is 56 °C, but drops sharply to 42 °C in the FZ (Figure 32). During the CPT measurement, a static potential of 0.75 V (SCE) is applied to the specimen while the electrolyte temperature is increased continuously at 1 °C/min. CPT is taken as the current density reaches 100 μA/cm^2^. By examining CPT specimens using optical microscopy and SEM, pits are found to locate preferentially in ferritic grains of weld metal (Figure 33). PWHT also restores a favorable α:γ ratio, thereby improving the pitting corrosion resistance of S31803 weldment accordingly.

**Figure 34:**
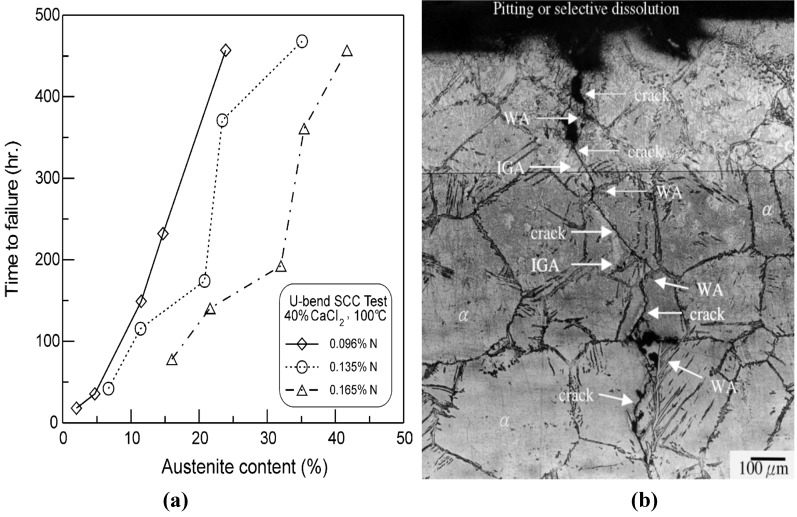
(**a**) Effect of austenite content on time-to-failure of simulated HAZs with various N contents in 40 wt% CaCl_2_ solution at 100 °C; (**b**) Cross-sectional optical micrograph showing crack propagation path in the HAZ of DSS 2205 with 0.165N under a simulated cooling rate of ~60 °C/s after U-bend SCC testing, reprinted with permission from [92]. Copyright 2012 Elsevier.

The HAZ often experiences thermal cycles with temperatures ranging from ambient up to the melting point in regions close to the weld. Furthermore, the heating and cooling rates may vary markedly with heat input, structural dimension, and position relative to the weld [5]. Therefore, the actual HAZ exhibits a complex mix of microstructures within a small volume next to the FZ. Moreover, because the width of HAZ is narrow, it is rather difficult to analyze the effect of a characteristic microstructure on stress corrosion using the true weldment. In this regard, the Gleeble thermo-mechanical simulator allows the simulation of various microstructures developed in HAZ at designed thermal cycles via resistive heating of metal samples [91]. For example, Liou *et al.* [92] used a Gleeble thermo-mechanical simulator for the welding HAZ simulation of DSS 2205 containing different N contents. The simulated specimens were subjected to U-bend SCC tests in 40 wt% CaCl_2_ solution at 100 °C. They reported that the GBA, WA and IGA were formed in the HAZ, and their contents varied with cooling rates and N contents in DSS 2205. The HAZ sample with 0.165N exhibited higher austenite and fewer Cr_2_N contents, leading to better SCC resistance (Figure 34a). In addition, pitting corrosion assisted the crack initiation, while the types and amounts of reformed austenite in the HAZ affected the mode of crack propagation. The GBA was found to promote intergranular stress corrosion cracking, but WA and IGA exhibited a beneficial effect on stress corrosion by deviating the crack propagation path (Figure 34b). Recently, Singh Raman demonstrated that threshold stress intensity factor for SCC (*K*_ISCC_), determined from fracture mechanics testing, is an important parameter in design and prediction of life of welded components [93]. *K*_ISCC_ is a stress intensity limit below which the crack cannot propagate in a corrosive solution. Determination of *K*_ISCC_ of narrow HAZ of real welds is difficult with conventional techniques. They recommended circumferential notch tensile technique instead. Alternatively, Gleeble simulator can be used to prepare HAZ samples for the study of crack extension in weldments of DSSs.

For welded DSSs, cracking and failure often initiated in fusion zone due to the presence of internal defects, chemical inhomogeneity and microstructural modification. Young *et al.* [94] reported that laser-welded DSS 2205 is susceptible to embrittlement in gaseous hydrogen. The hydrogen embrittlement susceptibility was correlated with the microstructures of fusion zone. The susceptibility decreased with increasing austenite content in the weld metal [94]. As mentioned above, sigma phase and chromium nitrides can be induced in the weld metals of DSSs [5,80,81,87,88]. These microstructural features deteriorate the corrosion resistance of weldments, resulting in IGC and pitting corrosion. Furthermore, pitting can assist initiation of fine cracks in DSSs under tensile stress, leading to stress corrosion in DSSs [69,72]. It is considered that pitting initiated in the welds can cause environmental cracking susceptibility largely, leading to catastrophic failure of weldments, particularly in marine environments.

## 5. Conclusions

Duplex stainless steels contain Cr, Mo and N alloying elements exhibiting good corrosion resistance in acidic, caustic and marine environments. However, intermetallic phase formed at 700–900 °C and Cr-rich α’-precipitates formed at 350–550 °C are detrimental to the corrosion resistance of DSSs, especially for highly alloyed steels. The formation of these phases depletes Cr or Cr/Mo content in the matrix adjacent to the precipitates, leading to IGC, pitting corrosion and SCC. Such phases are induced in DSSs during the fabrication, improper heat treatment and welding process. Therefore, care must be taken in the alloy design of modern DSSs to ensure optimal loading levels of alloying elements with a stable duplex structure. A balance between the chemical composition and corrosion resistance in DSSs must be maintained to achieve structural integrity.
